# The determinants of healthcare utilisation in regional, rural and remote South Australia: A cross‐sectional study

**DOI:** 10.1111/hsc.13894

**Published:** 2022-06-30

**Authors:** Matthew J. Leach, Kate Gunn, Kuda Muyambi

**Affiliations:** ^1^ National Centre for Naturopathic Medicine Southern Cross University East Lismore New South Wales Australia; ^2^ Department of Rural Health University of South Australia Whyalla Norrie South Australia Australia

**Keywords:** determinants, health services research, help‐seeking behaviour, rural health services, service utilisation

## Abstract

Accessibility of health services outside metropolitan centres in Australia is sub‐optimal. Recognising the barriers and enablers of healthcare access in rural, remote and regional settings is necessary to improving health service access in these disadvantaged populations. Accordingly, this study aimed to examine the determinants of healthcare use in rural, remote and regional South Australia. Cross‐sectional survey data were collected from adults living outside metropolitan Adelaide in South Australia between April 2017 and March 2018. Using a multi‐modal recruitment campaign, eligible adults were invited to complete the 44‐item consumer utilisation, expectations and experiences of healthcare instrument. Independent predictors of health service utilisation (dependent variable) were determined using negative binomial regression. The questionnaire was completed by 3926 predominantly female (52.5%) adults aged ≥50 years (56.7%), residing in regional South Australia (84.5%). Fifteen independent variables were significantly associated with health service utilisation using univariate analyses. Using negative binomial regression analysis, two predisposing factors (sex, remoteness), three enabling factors (income, health literacy, employment), two need factors (health rating, multimorbidity) and two personal health practices (alcohol, diet) were independently and significantly associated with healthcare use. Female sex (OR = 1.436, *p* < 0.001), good/excellent health rating (OR = 0.589, *p* < 0.001) and high multimorbidity (OR = 1.408, *p* < 0.001) were the strongest predictors of health service use. These findings will help inform the development of targeted health promotion and service engagement strategies for regional populations, which in addition to addressing workforce shortages, may help address inequity in health outcomes, particularly for groups engaging with regional healthcare services infrequently.

**What is known about this topic?**

- Myriad factors havebeen shown to impact access to, and utilisation of health care services.
- Understandingand managing these determinants is critical to addressing the unmet health careneeds of communities.
- The determinantsof health care access/utilisation in regional South Australia is currently poorlyunderstood.

**What this paper adds?**

- Our studyidentified myriad predisposing, enabling and need factors, and personal healthpractices that predicted health service utilisation in this population.
- The range offactors impacting health service use indicate this phenomenon is more complexthan previously anticipated.
- Understanding this complexity is critical todeveloping effective policies and strategies to improve health care access/utilisationin this population.

## 1 BACKGROUND

Access to healthcare systems that facilitate “equality of opportunity for people to enjoy the highest attainable health” is a right of every human being (Committee on Economic Social and Cultural Rights, 2000). Within this context, access refers not only to the physical availability of health services (i.e. services can be reached within a timely manner) but also their approachability (i.e. services are visible and transparent), acceptability (i.e. services are inclusive and accommodating of consumer differences), affordability (i.e. services are neither cost nor time prohibitive), appropriateness (i.e. services are able to adequately address consumer/community need) and subsequent utilisation (or realised access) (Goudge et al., 2009; Levesque et al., 2013; Russell et al., 2013). Each of the elements on this access‐utilisation continuum are necessary to ensure that health service provision is commensurate with consumer need (Levesque et al., 2013; Thomas et al., 2015).

Health service access/utilisation is more than just a structural issue; rather, it represents a complex interaction between systems, organisations, providers, consumers and the environment (Levesque et al., 2013). From the consumer's perspective, factors such as health literacy, ability to pay, health behaviour, health beliefs and trust in providers, influence the extent to which a person can effectively engage with the healthcare system (Derose et al., 2011). Environmental factors that impact engagement include access to transportation, living situation, and geographical location (Derose et al., 2011). Where populations are exposed to multiple structural, environmental, and consumer‐related barriers to healthcare access, there is likely to be a reduction in service utilisation, and subsequently, considerable unmet healthcare need (Corscadden et al., 2017; Russell et al., 2013). This is certainly apparent in rural, remote and regional locations, where geographical isolation, stoicism and healthcare avoidance behaviours are evident (Bourke et al., 2012), and availability, approachability, acceptability, affordability, appropriateness and utilisation of health services are often less than optimal (Leach et al., 2021; Thomas et al., 2015).

An important first step to improving access to healthcare in rural, remote and regional populations (hereon referred to as ‘regional’) is recognising the factors that facilitate and hinder healthcare access/utilisation in these populations. These factors—referred to as determinants of healthcare utilisation—are not only numerous and complex but also vary in their conceptualisation and operationalisation. One framework that has been well used over the past five decades, across diverse healthcare settings including regional settings (Li et al., 2016; Shah et al., 2014), and is clear and concise in its conceptualisation of these determinants, is Andersen's behavioural model of healthcare utilisation (Andersen & Davidson, 2007).

Andersen's model posits that healthcare access/utilisation is influenced by four types of factors (domains): predisposing (e.g. education, region of residence), enabling (e.g. income, employment status), need (e.g. health status, morbidity) and personal health practice (e.g. alcohol consumption, smoking status) factors (Andersen & Davidson, 2007). Using Andersen's model to understand the determinants of healthcare utilisation in regional populations may help facilitate improvements in health service access/use within these regions, by informing the development of appropriate and targeted health services planning activities, health policies and needs‐based resource allocation.

Given that regional communities are not homogenous, each community is likely to encounter different healthcare needs, challenges and opportunities. Accordingly, any health workforce/services planning activities conducted in these regions should be undertaken at a level that is likely to generate findings that are meaningful and translatable to these communities. Such activity should also take into account constraints associated with jurisdictional boundaries (e.g. health system borders). Taking these factors into consideration, we focussed our attention on a group of communities that were serviced by the same state‐based health system and governed by the same health policies. We also selected a region where healthcare access/utilisation was problematic (i.e. there was considerable spatial isolation), there was known to be a high level of unmet healthcare needs, and the factors impacting healthcare access/utilisation had not been rigorously examined.

Regional South Australia represents one of the least densely populated regions in the world (Population of Australia, 2019), where rates of chronic disease, co‐morbidity, and psychological distress are among the highest of any Australian State or Territory (Australian Institute of Health and Welfare, 2016). And yet, the predictors of healthcare use in this region have received little attention to date. To address this knowledge gap, we aimed to examine the determinants of healthcare utilisation in regional South Australia.

## 2 METHODS

### 2.1 Study design

The Regional South Australia Health (RESONATE) project was a cross‐sectional study designed to explore the healthcare needs and behaviours of the remote, rural and regional South Australian population. The study was developed to address eight primary objectives, which are described in detail elsewhere (Leach et al., 2018). This paper responds to the eighth objective of RESONATE: to identify the determinants of health service utilisation among adults living in regional South Australia. The specific question that this research intended to answer was: What predisposing, enabling, need and personal health practice factors predict health service use in a sample of regional South Australians?

Health service use (dependent variable) was defined as the sum number of visits to all healthcare providers in the preceding 12 months. This included visits to a nursing/midwifery (e.g. nurse, nurse practitioner, midwife), complementary medicine (e.g. acupuncturist, aromatherapist, Ayurvedic practitioner, chiropractor, herbalist, homeopath, kinesiologist, massage therapist, naturopath, osteopath, reflexologist, reiki practitioner, shiatsu practitioner, tai chi instructor, traditional Chinese medicine practitioner, yoga instructor), allied health (e.g. dietician/nutritionist, exercise physiologist, optician/optometrist, occupational therapist, pharmacist, physiotherapist, podiatrist, psychologist/counsellor, social worker, speech pathologist) or medical (e.g. general practitioner, medical specialist, dentist) professional. We examined healthcare use across all health providers, rather than by particular disciplines or professional groupings, to capture a ‘whole of system’ perspective of the phenomenon.

### 2.2 Participants

Adult residents of regional, rural, remote or very remote South Australia, who had used any healthcare service or health intervention in the preceding 12 months, and had internet access or a fixed address (to enable the questionnaire to be completed online or to be posted, respectively) were eligible for inclusion in the study. Residents who had severe cognitive or visual impairment, or were unable to provide informed consent, were ineligible to participate. Based on a ±3% margin of error, 99% confidence level, and a target population of 290,290 adults (Australian Bureau of Statistics, 2018a), we required a sample size of at least 1832 people to identify health service use at any prevalence rate (i.e. frequency of the outcome in the population = 50%).

### 2.3 Data collection

Participants were recruited using non‐probability sampling and an extensive, multi‐modal marketing campaign. Recruitment was undertaken between April 2017 and March 2018 and included the use of print media (e.g. newspaper articles, residential mailbox drop across regional South Australia), online media (e.g. organisational email blasts, development of a dedicated project website), social media (e.g. Instagram and Twitter posts, Facebook advertisements), broadcast media (e.g. radio interviews, television classified advertisements) and community engagement activities (e.g. attendance at community/public events, meetings/presentations with regional stakeholder groups). A detailed description of the recruitment campaign is reported elsewhere (Leach et al., 2018). All recruitment activities directed participants to either call a toll‐free telephone number (to acquire a print‐copy of the questionnaire) or visit the project website (where they could complete the questionnaire online, via the SurveyMonkey™ platform). Questionnaires completed in print‐copy were returned by reply‐paid mail, and on receipt, were manually entered into the online survey platform by the research team.

### 2.4 Questionnaire

Participants were invited to complete the consumer utilisation, expectations, and experiences of healthcare instrument (CONVERSATIONS)—a questionnaire specifically designed to measure healthcare need, health service utilisation, and the determinants of healthcare use (Leach et al., 2018). This self‐administered, multidimensional questionnaire comprises 44 items, divided into five segments: lifestyle and health status (10 items), preferences concerning health service mix (1 item), use/attitude/experience/satisfaction of conventional health services (8 items), use/attitude/experience/satisfaction of complementary health/self‐prescribed services (9 items) and demographic characteristics (16 items). Psychometric evaluation of CONVERSATIONS has shown the instrument to have acceptable internal reliability, good test–retest reliability, a high degree of acceptability and good content validity. A more detailed narrative of the development and validation of CONVERSATIONS is included in the study protocol (Leach et al., 2018).

### 2.5 Exposures

The four exposures in this study reflect the four determinants of healthcare use described in Andersen's behavioural model of healthcare utilisation (Andersen & Davidson, 2007):

- *Predisposing factors*: These preceding conditions predispose an individual to use a health service (Leach et al., 2018). These conditions include age (i.e. 18–29 years, 30–49 years, 50–69 years, 70 years and over), sex (i.e. male vs. female), country of birth (i.e. Australia vs. other country), remoteness area (using the Australian Statistical Geography Standard (Australian Bureau of Statistics, 2018b) classes of remoteness; i.e. inner regional [RA2], outer regional [RA3], remote [RA4] and very remote [RA5]), marital status (i.e. not married vs. married), religiosity (i.e. non‐religious vs. religious), highest level of education (i.e. no/primary/secondary education vs. tertiary education), number of dependent children (i.e. no children vs. one or more children) and primary caregiver role (i.e. not a primary caregiver vs. primary caregiver).
- *Enabling factors*: These factors foster or impede health service use (Leach et al., 2018), including: gross annual household income (i.e. AU$0–$29,999, AU$30,000–$59,999, AU$60,000–$89,999, AU$90,000 or more), employment status (i.e. unemployed/retired vs. employed), English language proficiency (i.e. not at all/not well vs. well/very well) and health literacy (i.e. needs assistance none of the time vs. needs assistance some/most/all of the time).
- *Need factors*: These factors represent a potential or actual need for healthcare (Leach et al., 2018). These need factors were measured using overall health rating (i.e. perception of one's current state of health on a single‐item, 5‐point scale, with responses ranging from fair to excellent), and level of multimorbidity (i.e. the presence of no chronic disease, one chronic disease, two chronic diseases or three or more chronic diseases), as defined by the Family Medicine Research Centre (O'Halloran et al., 2004).
- *Personal health practice factors*: These factors represent lifestyle activities that impact health status, and the future need for healthcare (Leach et al., 2018), including:
  - alcohol consumption (reported as level of compliance; i.e. non‐compliant [>2 standard drinks/day] vs. compliant [0–2 standard drinks/day]; as measured by the 2009 NHMRC guidelines for the consumption of alcohol; National Health & Medical Research Council, 2009),
  - smoking status (i.e. non‐smoker vs. current smoker),
  - physical activity (reported as compliance with WHO physical activity guidelines; i.e. non‐compliant [<300 min of moderate physical activity or <150 min of vigorous physical activity or an equivalent combination per week] vs. compliant [≥300 min of moderate physical activity or ≥150 min of vigorous physical activity or an equivalent combination per week]; as measured by the 2‐item Nordic Physical Activity Questionnaire [NPAQ]‐short; Danquah et al., 2018),
  - diet quality (reported as level of compliance with Australian dietary guidelines; i.e. low compliance [score = 0–74.9] vs. high‐compliance [score = 75–150] with recommended daily fruit, vegetable and all food group consumption; as measured by the 15‐item dietary guideline index; McNaughton et al., 2008), and
  - risk of lifestyle‐associated all‐cause mortality (reported as no risk [no risk factors] vs. elevated risk [1 or more risk factors]; as measured by the 6‐item Lifestyle Risk Index; Ding et al., 2015).

### 2.6 Data analysis

Data were analysed using IBM® SPSS® Statistics v.25.0. Konstan et al. (2005) deduplication procedure for online surveys was used to identify and manage duplicate entries. Any data omitted from non‐forced response items (i.e. religion, household income) were reported as ‘missing’. To account for the non‐probability sampling strategy, we adjusted the survey sample distribution by applying weights to the sex, age (i.e. by 5‐year age group), and location distribution (i.e. by ABS statistical area 3 level) of the regional SA population. Weights were derived from 2016 Australian population census data (Australian Bureau of Statistics, 2019). The dependent variable (i.e. frequency of healthcare utilisation over the previous 12 months) was log‐transformed to ensure data followed a near‐normal distribution. Univariate analyses (using independent samples *t*‐tests for comparisons between two sub‐groups, and ANOVA for comparisons between three or more sub‐groups) were performed to identify differences in healthcare utilisation between sub‐groups (e.g. female vs. male) of each variable (e.g. sex). Variables demonstrating a statistically significant (*p* < 0.05) difference in healthcare utilisation were included in the regression models. We used negative binomial regression to examine the association between the dependent variable—healthcare utilisation (i.e. number of visits with any healthcare provider)—and the independent variables listed within each of the four domains of Andersen's model (i.e. predisposing, enabling, need and personal health practice factors). A two‐sided *p*‐value <0.05 was considered statistically significant. To ensure the assumptions for negative binomial regression were not violated, we tested for normality of distribution (using probability–probability plots), homoscedasticity (using scatterplots), and multicollinearity (using variance inflation factors).

### 2.7 Ethics

This study was reviewed and approved by the Human Research Ethics Committee of the University of South Australia (Protocol ID: 0000034611). All participants were provided with a copy of the study information sheet and were required to provide informed consent before commencing the survey. Participants were anonymous, and they could choose to opt‐in/opt‐out of the study at their discretion, without consequence.

## 3 RESULTS

The questionnaire was completed by 3926 adults. The effective sample size (after adjusting for sex, age and location distribution) was 3743. Calculation of the survey response rate was not possible as the denominator (i.e. population reached) was not known.

### 3.1 Demographic characteristics

Participants (weighted cases) predominantly resided in inner or outer regional areas (RA2‐3) of South Australia (84.5%; Table 1). Most were born in Australia (84.2%) and reported a high level of English language proficiency (99.7% reporting speaking English well or very well) and health literacy (with 70.3% requiring no assistance with understanding health information). Although most participants rated their overall health as good, very good or excellent (72.6%), almost two‐thirds (58.8%) were living with two or more chronic diseases. Similarly, while most participants were non‐smokers (90.8%) and were compliant with Australian dietary guidelines (97.1%), NHMRC alcohol consumption guidelines (89.1%), and WHO physical activity guidelines (70.3%), half (52.5%) had an elevated risk of lifestyle‐associated all‐cause mortality. The sum number of visits to all healthcare providers in the preceding 12 months, as reported by participants, ranged from 0 to 520, with a median of 17 (IQR 9,33) visits.

**TABLE 1:** Characteristics of study participants

Determinant	Sub‐group	Weighted (*n* = 3743), *n* (%)	Unweighted (*n* = 3926), *n* (%)
Predisposing factors			
Age	18–29 years	502 (13.4)	230 (5.9)
	30–49 years	1117 (29.8)	869 (22.1)
	50–69 years	1404 (37.5)	1862 (47.4)
	70 years and over	720 (19.2)	965 (24.6)
Sex	Male	1777 (47.5)	1266 (32.3)
	Female	1966 (52.5)	2660 (67.7)
Country of birth	Other country	590 (15.8)	634 (16.1)
	Australia	3153 (84.2)	3292 (83.9)
ASGC remoteness area classification	Inner regional (RA 2)	1239 (33.1)	1206 (30.7)
	Outer regional (RA 3)	1922 (51.4)	2027 (51.6)
	Remote (RA 4)	494 (13.2)	576 (14.7)
	Very remote (RA 5)	88 (2.4)	117 (3.0)
Religiosity	Non‐religious	1640 (43.8)	1655 (42.2)
	Religious	2103 (56.2)	2271 (57.8)
Marital status	Not married	1644 (43.9)	1535 (39.1)
	Married	2099 (56.1)	2391 (60.9)
Highest education	None, primary or secondary education	1641 (43.8)	1811 (46.1)
	Tertiary education	2102 (56.2)	2115 (53.9)
Number of dependent children	No children	2298 (61.4)	2501 (63.7)
	One or more children	1445 (38.6)	1425 (36.3)
Primary caregiver status	Not a primary caregiver	3315 (88.6)	3453 (88.0)
	A primary caregiver	428 (11.4)	473 (12.0)
Enabling factors			
Annual gross household income (AUD)	$0–$29,999	755 (20.2)	812 (20.7)
	$30,000–$59,999	989 (26.4)	1108 (28.2)
	$60,000–$89,999	659 (17.6)	625 (15.9)
	$90,000 or more	980 (26.2)	941 (24.0)
	Missing	360 (9.6)	440 (11.2)
Employment status	Unemployed/retired	1800 (48.1)	2149 (54.7)
	Employed	1943 (51.9)	1777 (45.3)
English language proficiency	Not at all/not well	10 (0.3)	7 (0.2)
	Well/very well	3733 (99.7)	3919 (99.8)
Health literacy	Needs assistance none of the time	2633 (70.3)	2871 (73.1)
	Needs assistance some of the time	987 (26.4)	968 (24.7)
	Needs assistance most of the time	103 (2.8)	62 (1.6)
	Needs assistance all of the time	20 (0.5)	25 (0.6)
Need factors			
Overall health rating	Poor/fair	1025 (27.4)	1042 (26.5)
	Good	1187 (31.7)	1289 (32.8)
	Very good/excellent	1531 (40.9)	1595 (40.6)
Multimorbidity index	Living with no chronic disease	756 (20.2)	733 (18.7)
	Living with 1 chronic disease	785 (21.0)	801 (20.4)
	Living with 2 chronic diseases	786 (21.0)	804 (20.5)
	Living with 3+ chronic diseases	1416 (37.8)	1588 (40.4)
Personal health practices			
Compliance with NHMRC alcohol consumption guidelines (lifetime risk)	Non‐compliant	409 (10.9)	351 (8.9)
	Compliant	3334 (89.1)	3575 (91.1)
Smoking status	Non‐smoker	3399 (90.8)	3596 (91.6)
	Smoker	344 (9.2)	330 (8.4)
Compliance with WHO physical activity guidelines	Non‐compliant	1111 (29.7)	1138 (29.0)
	Compliant	2632 (70.3)	2788 (71.0)
Compliance with Australian dietary guidelines	Low compliance	108 (2.9)	87 (2.2)
	High compliance	3635 (97.1)	3839 (97.8)
Risk of lifestyle‐associated all‐cause mortality	No risk (no risk factors)	1777 (47.5)	2040 (52.0)
	Elevated risk (one or more risk factors)	1966 (52.5)	1886 (48.0)

Abbreviations: ASGC, Australian Standard Geographical Classification; AUD, Australian dollars; NHMRC, National Health & Medical Research Council; RA, remoteness area; WHO, World Health Organisation.

### 3.2 Predisposing factors

Univariate analyses (of weighted cases) found five of the nine predisposing factors were statistically significantly associated with healthcare use (Table 2). Significantly higher rates of healthcare utilisation were evident among female participants, as well as those that had completed none/primary/secondary education, identified as religious, were born in Australia and resided in inner regional locations.

**TABLE 2:** Frequency of visits with healthcare providers over the past 12 months, by predisposing, enabling, need and personal health practice factors

Determinant	Sub‐group	Weighted (*n* = 3743)		Unweighted (*n* = 3926)	
		Median number of visits (IQR)	*p* value (^b^)	Median number of visits (IQR)	*p* value (^b^)
Predisposing factors					
Age	18–29 years^a^	15 (9,29)	0.055	18 (11,36)	0.993
	30–49 years	16 (8,34)	*(F = 2.539)*	19 (10,36)	*(F = 0.030)*
	50–69 years	18 (9,34)		19 (10,34)	
	70 years and over	18 (9,33)		19 (10,34)	
Sex	Male^a^	14 (7,28)	**<0.001**	17 (8,30)	**<0.001**
	Female	20 (11,37)	*(t = −9.194)*	20 (11,37)	*(t = −5.876)*
Country of birth	Other country^a^	16 (9,28)	**0.012**	17 (9,32)	**0.030**
	Australia	18 (9,34)	*(t = −2.525)*	19 (10,35)	*(t = −2.175)*
ASGC remoteness area classification	Inner regional (RA 2)^a^	19 (9,37)	**0.009**	20 (10,37)	**0.019**
	Outer regional (RA 3)	17 (9,32)	*(F = 3.825)*	19 (10,34)	*(F = 3.315)*
	Remote (RA 4)	17 (9,30)		18 (9,21)	
	Very remote (RA 5)	16 (7,27)		19 (9,29)	
Religiosity	Non‐religious^a^	16 (8,33)	**0.015**	18 (9,33)	**0.008**
	Religious	18 (9,33)	*(t = −2.426)*	20 (10,35)	*(t = −2.672)*
Marital status	Not married^a^	18 (9,35)	0.245	20 (10,36)	0.256
	Married	17 (9,31)	*(t = 1.163)*	18 (10,33)	*(t = 1.136)*
Highest education	None, primary or secondary education^a^	18 (9,34)	**0.002**	19 (10,35)	0.526
	Tertiary education	16 (9,32)	*(t = −3.085)*	19 (10,34)	*(t = 0.635)*
Number of dependent children	No children^a^	18 (9,34)	0.111	19 (10,35)	0.399
	One or more children	16 (9,31)	*(t = −1.592)*	19 (10,34)	*(t = 0.843)*
Primary caregiver status	Not a primary caregiver^a^	17 (9,33)	0.619	19 (10,35)	0.981
	A primary caregiver	17 (9,31)	*(t = −0.497)*	19 (11,33)	*(t = −0.024)*
Enabling factors					
Annual gross household income (AUD)	$0–$29,999^a^	21 (10,37)	**<0.001**	21 (12,37)	**0.001**
	$30,000–$59,999	18 (9,33)	*(F = 9.808)*	19 (10,34)	*(F = 5.583)*
	$60,000–$89,999	16 (10,29)		19 (10,32)	
	$90,000 or more	15 (7,31)		18 (9,34)	
Employment status	Unemployed/retired^a^	21 (10,37)	**<0.001**	21 (11,37)	**<0.001**
	Employed	14 (8,29)	*(t = 8.431)*	17 (9,31)	*(t = 6.187)*
English language proficiency	Not at all/not well^a^	12 (7,65)	0.231	9 (3,18)	0.354
	Well/very well	17 (9,33)	*(t = −1.296)*	19 (10,34)	*(t = −1.020)*
Health literacy	Needs assistance none of the time^a^	16 (8,31)	**<0.001**	18 (9,33)	**<0.001**
	Needs assistance some of the time	21 (11,35)	*(F = 18.895)*	21 (11,39)	*(F = 22.859)*
	Needs assistance most of the time	21 (11,39)		27 (14,48)	
	Needs assistance all of the time	41 (24,94)		34 (22,67)	
Need factors					
Overall health rating	Poor/fair^a^	28 (15,47)	**<0.001**	29 (17,50)	**<0.001**
	Good	17 (9,30)	*(F = 131.193)*	18 (10,33)	*(F = 122.079)*
	Very good/excellent	13 (7,25)		15 (8,26)	
Multimorbidity index	Living with no chronic diseases^a^	11 (6,22)	**<0.001**	14 (6,25)	**<0.001**
	Living with 1 chronic disease	14 (7,25)	*(F = 51.645)*	15 (8,27)	*(F = 45.227)*
	Living with 2 chronic diseases	19 (10,34)		19 (10,31)	
	Living with 3+ chronic diseases	23 (12,42)		25 (14,43)	
Personal health practices					
Compliance with NHMRC alcohol consumption guidelines (lifetime risk)	Non‐compliant^a^	13 (7,25)	**<0.001**	15 (8,31)	**0.001**
	Compliant	18 (9,34)	*(t = −4.985)*	19 (10,35)	*(t = −3.498)*
Smoking status	Non‐smoker^a^	17 (9,32)	**0.043**	19 (10,34)	**0.169**
	Smoker	19 (8,36)	*(t = −2.026)*	20 (10,36)	*(t = −1.377)*
Compliance with WHO physical activity guidelines	Non‐compliant^a^	20 (9,35)	**0.001**	20 (10,36)	**0.016**
	Compliant	16 (9,32)	*(t = 3.187)*	19 (10,34)	*(t = 2.401)*
Compliance with Australian dietary guidelines	Low compliance^a^	11 (10,31)	**0.031**	17 (9,41)	0.694
	High compliance	18 (9,33)	*(t = −2.191)*	19 (10,34)	*(t = −0.395)*
Risk of lifestyle‐associated all‐cause mortality	No risk (no risk factors)^a^	18 (9,31)	**0.015**	18 (10,32)	**0.005**
	Elevated risk (1 or more risk factors)	17 (9,35)	*(t = −2.439)*	20 (10,36)	*(t = −2.814)*

Abbreviations: ASGC, Australian Standard Geographical Classification; AUD, Australian dollars; IQR, interquartile range; NHMRC, National Health & Medical Research Council; RA, remoteness area; WHO, World Health Organisation.

^a^ Reference group.

^b^ Difference in the mean number of healthcare provider visits between subgroups (using independent‐sample *t*‐tests of log_10_ transformed weighted data for comparisons between two groups, and ANOVA for comparisons between three or more groups).

Statistically significant *p* values are in bold. Test statistics are in italics.

### 3.3 Enabling factors

Three of the four enabling factors were shown to be statistically significantly associated with healthcare use (Table 2). Participants that were unemployed or retired, had suboptimal health literacy (i.e. required assistance with understanding health information some/most/all the time) and reported an annual gross household income of less than AU$60,000 had significantly higher levels of healthcare utilisation than their subgroup counterparts.

### 3.4 Need factors

Univariate analyses found both need factors to be statistically significantly associated with healthcare use (Table 2). The frequency of healthcare provider visits over the past 12 months was significantly greater among participants living with multimorbidity (i.e. two or more chronic conditions), and those rating their overall health as fair or poor.

### 3.5 Personal health practices

Of the five personal health practices examined, all were shown to be statistically significantly associated with healthcare use (Table 2). Significantly higher rates of healthcare utilisation were found for smokers, those reporting a lower level of compliance with WHO physical activity guidelines, as well as those demonstrating a higher level of compliance with NHMRC alcohol consumption guidelines and Australian dietary guidelines, and those with no risk of lifestyle‐associated all‐cause mortality.

### 3.6 Predictors of health service utilisation

The 15 independent variables that were significantly associated with health service utilisation were entered into a negative binomial regression model (Table 3). The regression model was found to be statistically significantly better than the intercept‐only model at predicting health service utilisation (Omnibus test of model coefficient: χ2[24] = 516.616, *p* < 0.001). Health service utilisation was shown to be predicted by all but six independent variables (i.e. country of birth, religiosity, highest level of education, smoking status, compliance with WHO physical activity guidelines, and risk of lifestyle‐associated all‐cause mortality; Table 3).

**TABLE 3:** Results of negative binomial regression showing the effect of predisposing, enabling, need and personal health practice factors on health service utilisation

Determinant	Sub‐group	Weighted (*n* = 3743)			Unweighted (*n* = 3926)		
		Adjusted odds ratio	95% confidence interval	*p* value	Adjusted odds ratio	95% confidence interval	*p* value
Predisposing factors							
Sex	Male	Reference					
	Female	1.436	1.336–1.543	**<0.001**	1.337	1.238–1.445	**<0.001**
Country of birth	Other country	Reference					
	Australia	1.014	0.923–1.114	0.778	1.008	0.990–1.196	0.081
ASGC remoteness area classification	Inner regional (RA 2)	Reference					
	Outer regional (RA 3)	0.931	0.863–1.004	0.064	0.932	0.862–1.007	0.075
	Remote (RA 4)	0.886	0.793–0.989	**0.031**	0.853	0.764–0.952	**0.005**
	Very remote (RA 5)	0.775	0.603–0.996	**0.046**	0.758	0.612–0.940	**0.011**
Religiosity	Non‐religious	Reference					
	Religious	1.066	0.995–1.143	0.069	1.099	1.025–1.178	**0.008**
Highest education	None, primary or secondary education	Reference					
	Tertiary education	1.011	0.939–1.088	0.771	1.040	0.967–1.118	0.291
Enabling factors							
Annual gross household income (AUD)	$0–$29,999	Reference					
	$30,000–$59,999	1.050	0.955–1.156	0.313	1.007	0.916–1.106	0.887
	$60,000–$89,999	1.104	0.983–1.241	0.094	1.051	0.936–1.181	0.400
	$90,000 or more	1.138	1.015–1.275	**0.026**	1.119	0.999–1.254	0.052
Employment status	Unemployed /retired	Reference					
	Employed	0.888	0.818–0.964	**0.005**	0.881	0.810–0.958	**0.003**
Health literacy	Needs assistance none of the time	Reference					
	Needs assistance some of the time	1.213	1.119–1.314	**<0.001**	1.196	1.102–1.298	**<0.001**
	Needs assistance most of the time	1.216	0.972–1.521	0.087	1.173	0.894–1.538	0.250
	Needs assistance all of the time	1.543	0.986–2.416	0.058	1.774	1.148–2.743	**0.010**
Need factors							
Overall health rating	Poor/fair	Reference					
	Good	0.644	0.585–0.709	**<0.001**	0.651	0.592–0.716	**<0.001**
	Very good/excellent	0.589	0.532–0.652	**<0.001**	0.613	0.555–0.678	**<0.001**
Multimorbidity index	Living with no chronic diseases	Reference					
	Living with 1 chronic disease	1.056	0.948–1.177	0.319	1.037	0.928–1.159	0.523
	Living with 2 chronic diseases	1.218	1.092–1.359	**<0.001**	1.118	1.000–1.251	0.050
	Living with 3+ chronic diseases	1.408	1.267–1.565	**<0.001**	1.358	1.222–1.510	**<0.001**
Personal health practices							
Compliance with NHMRC alcohol consumption guidelines (lifetime risk)	Non‐compliant	Reference					
	Compliant	1.162	1.032–1.308	**0.013**	1.174	1.032–1.336	**0.015**
Smoking status	Non‐smoker	Reference					
	Smoker	0.952	0.840–1.078	0.436	0.908	0.798–1.034	0.146
Compliance with WHO physical activity guidelines	Non‐compliant	Reference					
	Compliant	1.039	0.958–1.126	0.358	1.070	0.985–1.162	0.109
Compliance with Australian dietary guidelines	Low compliance	Reference					
	High compliance	1.325	1.081–1.625	**0.007**	1.018	0.807–1.284	0.880
Risk of lifestyle‐associated all‐cause mortality	No risk (no risk factors)	Reference					
	Elevated risk (1 or more risk factors)	1.057	0.975–1.145	0.179	1.110	1.023–1.206	**0.013**

Abbreviations: ASGC, Australian Standard Geographical Classification; AUD, Australian dollars; NHMRC, National Health & Medical Research Council; WHO, World Health Organisation.

Statistically significant *p* values are in bold.

The need factors were found to be among the strongest predictors (based on adjusted odds ratios) of health service utilisation. The model indicated that participants with a high multimorbidity index (i.e. living with three or more chronic diseases, compared with no chronic diseases) had 40.8% (OR 1.408, 95% CI 1.267–1.565) greater odds of using health services, whereas those with a very good/excellent overall health rating (compared with a poor/fair health rating) had 41.1% (OR 0.589, 95% CI 0.532–0.652) lower odds of health service utilisation. In terms of predisposing factors, female participants had 43.6% greater odds (OR 1.436, 95% CI 1.336–1.543) of using health services compared with males; by contrast, participants living in a very remote area (compared with inner regional area) had 22.5% (OR 0.775, 95% CI 0.603–0.996) lower odds of health service utilisation. For personal health practices, participants compliant with NHMRC alcohol consumption guidelines and Australian dietary guidelines had 16.2% (OR 1.162, 95% CI 1.032–1.308) and 32.5% (OR 1.325, 95% CI 1.081–1.625) greater odds of health service utilisation, respectively, compared with non‐compliant participants. All enabling factors influenced health service utilisation to some extent. Healthcare utilisation among participants earning a high annual gross household income (i.e. A$90,000 or more vs. <A$30,000) and those employed (vs. unemployed/retired) increased by 13.8% (OR 1.138, 95% CI 1.015–1.275) and 11.2% (OR 0.888, 95% CI 0.818–0.964), respectively. Participants with moderate health literacy (i.e. needing assistance with health information some of the time vs. needing assistance none of the time), had 21.3% (OR 1.213, 95% CI 1.119–1.314) higher odds of using health services; low health literacy (i.e. needing assistance all of the time) did not significantly predict healthcare utilisation.

## 4 DISCUSSION

This study set out to identify the determinants of health service utilisation among adults living in regional South Australia. Eight factors were found to predict health service utilisation in this rural, regional and remote sample; including two predisposing factors, two enabling factors, two need factors and two personal health practices. The range of factors impacting health service use in this sample indicate that health service utilisation in regional communities is a complex issue.

Significantly higher rates of healthcare utilisation were evident among female participants, as well as those who resided in inner regional (as opposed to outer regional, remote or very remote) locations. Female gender has been widely found to be predictive of health service utilisation both in Australia (Mills et al., 2012; Parslow et al., 2004) and overseas (Fleury et al., 2012), as has rurality (Mills et al., 2012). These findings suggest that careful attention needs to be paid to ensuring that health services appropriately engage with, and are acceptable and accessible to, individuals living in regional settings, especially men. This is particularly important given that men are significantly more likely to die from preventable illness than women (Bots et al., 2017; Judd et al., 2008; Perez et al., 2020).

An interesting observation in this study was the absence of a statistically significant association between age and healthcare utilisation. Although this finding is contrary to the results of other research (where age and healthcare use have been shown to be positively correlated; Harrison et al., 2019; Recchia et al., 2022), the finding is not unique, with many studies reporting similarly divergent results (Agyemang‐Duah et al., 2020; Rana et al., 2019; Thompson et al., 2016). A possible explanation for our finding may relate to the sample itself. First, our sample comprised only of regional South Australians—a population shown in previous research to have high rates of stoicism and perceived need for self‐reliance—both of which have been identified as significant barriers to health service use in regional populations (Hull et al., 2017). Second, our sample (as reported elsewhere; Leach et al., 2020) had a high prevalence of mental health disorders relative to urban South Australia (not dissimilar to that reported internationally; Fu et al., 2018); these disorders are consistently reported as barriers to health service use (Leyva et al., 2020; Thompson et al., 2016). It is possible that these factors combined may have attenuated the association between age and healthcare use, further highlighting the complexity of healthcare utilisation in regional populations.

In terms of enabling factors, participants who were unemployed or retired or had an annual gross household income of greater than AU$90,000, reported significantly higher levels of healthcare utilisation than their subgroup counterparts. These findings are consistent with that reported in other Australian studies (Collie et al., 2021; Schlichthorst et al., 2016). Although these findings may appear contradictory, they can be elucidated to some extent through the lens of affordability. For instance, unemployed or retired persons in Australia may be eligible to receive national healthcare concessions to help reduce the costs of health services and medicines. By contrast, persons earning a high income may have the financial means to either pay for health services out‐of‐pocket or access private services covered by private health insurance (Carpenter et al., 2015; Gao et al., 2022). In either case, affordability appears to be an important predictor of healthcare access and utilisation.

Another determinant of healthcare utilisation is health need. Regional populations are disproportionally affected by chronic disease, and as such, have a high level of health need relative to urban populations (Hetzel et al., 2015; Leach et al., 2020). Indeed, our analyses revealed that multimorbidity (i.e. living with two or more chronic conditions) and fair/poor overall health were significant predictors of health service use. Findings from international studies corroborate these results (Fleury et al., 2010; Foguet‐Boreu et al., 2014; Glynn et al., 2011; Jardine et al., 2011). Although an integrative and multidisciplinary approach is preferential for the management of multimorbidity (Leach & Steel, 2018; Palmer et al., 2018; Smith et al., 2016), this approach may be somewhat problematic to implement in regional communities where access to diverse allied health services is generally poor. Thus, failing to address health workforce maldistribution in regional settings may essentially deny regional communities access to best practice care.

Personal health practices (including lifestyle behaviours) are closely correlated with the development and progression of chronic disease and multimorbidity (Freisling et al., 2020), as well as increased health service use, including hospitalisations (Arcury et al., 2005; Haapanen‐Niemi et al., 1999; Luben et al., 2020). While this was supported in univariate analyses, our regression analyses did not find lifestyle behaviours, such as smoking status and compliance with physical activity guidelines, to be significant predictors of healthcare utilisation in this regional South Australian sample. One explanation may relate to how some data were collected in the CONVERSATIONS instrument. For example, the smoking status item did not differentiate non‐smokers from former smokers. Given that former smokers are significantly more likely to use health services than non‐smokers and smokers (Kahende et al., 2009), it is likely that the non‐significant finding for smoking status may be the result of former smokers being conflated with non‐smokers. Similarly, the non‐significant finding for compliance with physical activity guidelines may relate to the dichotomisation of the variable, which may have contributed to some degree of confounding.

By contrast, our analyses revealed that participants reporting favourable personal health practices, including a high level of compliance with alcohol consumption guidelines and dietary guidelines, reported high levels of health service utilisation. Although this finding may seem counterintuitive, similar observations have been noted in other studies (Collins et al., 2011; Daya et al., 2020; Haapanen‐Niemi et al., 1999). Although it is not entirely clear why patient adherence to alcohol and dietary guidelines was associated with higher health service use, it may be that regular health service users were more exposed to contemporary health information on risks associated with alcohol consumption and poor dietary behaviour. Another possible explanation is that in light of the high prevalence of pre‐existing chronic disease and multimorbidity in the study sample, these health practices may simply reflect adaptive behaviours (i.e. consequential changes due to the diagnosis of a disease) rather than primary prevention behaviours. Either way, the findings point to several key areas requiring additional investigation; the findings of which may help further our understanding of the impact of health service access on the health practices of individuals living in regional communities.

The strengths of this study are the large sample size, the inclusion of participants from diverse remoteness categories, and the representativeness of the sample compared with regional South Australian population census data (e.g. sex [50% female], age [63% aged 30–69 years], country of birth [81% Australian‐born], marital status [50% married], and employment status [45% unemployed/retired]; ABS, 2019). However, there were some limitations. As this study relied on non‐probability sampling, the findings are susceptible to self‐selection bias. Similarly, as the survey was self‐administered, there is a potential for self‐reporting bias. Thus, the tendency of respondents to report favourably on habits such as dietary intake and alcohol consumption cannot be ruled out. Another consideration is that our study may have overlooked other pertinent factors impacting health‐seeking behaviour in this regional community. Thus, future work may need to consider looking beyond the variables measured in this study; for example, attitudinal influences on regional help‐seeking behaviour that may operate separately to health literacy (Dollman et al., 2021).

## 5 CONCLUSION

This study highlights a range of predisposing, enabling and need factors, and personal health practices that predict health service utilisation in rural, regional and remote South Australia. The findings have also alluded to several groups that may be susceptible to engaging less frequently with health services, including males, and those living in an outer regional, remote or very remote location. However, this phenomenon appears to be more complex than previously anticipated, with a wide range of factors contributing to health service utilisation in this population. Thus, further research is required to explore what the other predictors of health service use might be in this and other regional populations. At the very least, the findings from this research will help inform the development of policies and targeted strategies aimed at improving healthcare access, and reducing health inequities, in regional Australia.

## AUTHOR CONTRIBUTIONS

ML conceptualised the project. ML drafted the introduction, methods and results. KM, KG and ML drafted the discussion. All authors reviewed and edited the manuscript and approved the final manuscript.

## FUNDING INFORMATION

This study was supported by infrastructure provided by the Department of Rural Health, University of South Australia.

## CONFLICT OF INTEREST

None declared.

## Data Availability

The data that support the findings of this study are available from the corresponding author upon reasonable request.
